# Genetic Erosion in Captive Alpine Musk Deer Highlights the Challenges of Conserving Endangered Species in Closed Populations

**DOI:** 10.3390/ani15131827

**Published:** 2025-06-20

**Authors:** Han Jiang, Luyao Hai, Zhengwei Luo, Xianna Lan, Mi Zhou, Xinghu Qin, Defu Hu

**Affiliations:** 1 School of Ecology and Nature Conservation, Beijing Forestry University, Beijing 100083, China; 18813197210@163.com (H.J.); haily201756111@bjfu.edu.cn (L.H.); luozw114688@gmail.com (Z.L.); lanxianna66@bjfu.edu.cn (X.L.); 2 Sichuan Fengchun Musk Industry Group Co., Ltd., China Wildlife Conservation Association, Chengdu 610072, China; zhoumi1978@163.com

**Keywords:** captive alpine musk deer, mtDNA, haplotype, founding maternal lineage, genetic diversity

## Abstract

The alpine musk deer (*Moschus chrysogaster*) is a critically endangered species in China whose wild populations have precipitously declined due to habitat degradation and poaching. In response, China established the world’s first captive breeding facility for this species in 1990. Despite over three decades of closed breeding, the genetic consequences of long-term captivity remain unclear. Here, we present the first comprehensive assessment of mitochondrial genetic diversity in a captive population of 409 individuals, using three mitochondrial markers (D-loop, *Cytb*, and COI) and comparative data from wild conspecifics. Our results reveal a pronounced reduction in genetic diversity in the captive population compared to wild populations. Nucleotide diversity (π) and haplotype diversity (Hd) were consistently lower across all markers in captivity, with D-loop Hd = 0.639 and π = 0.01073. Further combined sequence analysis revealed a single dominant haplotype (Hap4) representing 56.99% of individuals, indicative of severe haplotype loss and homogenization. Although some haplotypes are shared with wild populations, captive populations exhibit strong genetic differentiation from wild populations, with the captive populations retaining only a limited fraction of the species’ maternal lineages. This pronounced genetic erosion driven by strong founder effects and genetic drift raises concerns about the viability and reintroduction success of this species. These findings highlight the inherent limitations of closed captive populations in preserving evolutionary potential and adaptive capacity. Our study emphasizes the urgent need for evidence-based genetic management, including founder augmentation and population exchange, to mitigate inbreeding and maintain genetic diversity.

## Simple Summary

Currently, there is limited research on the genetic diversity of the alpine musk deer (*Moschus chrysogaster*), with a lack of systematic studies. This research collected a total of 409 fecal samples from captive alpine musk deer in the Xinglong Mountains of Gansu Province. Mitochondrial DNA segments including D-loop, *Cytb*, and COI were sequenced and compared with available data from wild populations. The results showed significantly lower genetic diversity in the captive population, with reduced haplotype and nucleotide diversity, and the dominance of a single haplotype (Hap4, 56.99%). These findings indicate a strong founder effect and genetic drift due to long-term inbreeding and isolation. This study represents a comprehensive assessment of the genetic diversity of captive alpine musk deer to date and will contribute to the conservation and enhancement of genetic diversity in captive populations.

## 1. Introduction

The genetic diversity of wildlife is the result of the gradual accumulation of genetic mutations and recombinations over long evolutionary processes [1,2,3]. Genetic diversity not only reflects a species’ potential to adapt to environmental changes but also serves as a key indicator in the conservation biology of endangered species [4]. The richer the genetic diversity of a species, the greater its evolutionary potential and ability to adapt to changes in the environment. Traditionally, methods for assessing genetic diversity include cytological markers, biochemical markers, and molecular markers [5]. Among these, mitochondrial DNA (mtDNA) has been widely used in the genetic conservation studies of endangered animals due to its matrilineal inheritance, simple structure, high mutation rate, and rapid evolutionary speed [6,7,8].

D-Loop, *Cytb*, and COI are three distinct mtDNA fragments. The D-Loop is located in the control region of mtDNA and is a non-coding sequence, with a base substitution rate that is 5 to 10 times higher than that of coding regions. Therefore, the D-Loop is characterized by a rapid mutation rate and the ability to carry more recent and richer genetic information, meaning it is commonly used to assess population genetic diversity, genetic structure, and demographic history [6,9]. The variation rate of the cytochrome b (*Cytb*) sequence is moderate, allowing shorter DNA fragments to contain genetic information at the species level while exhibiting considerable variability. It is frequently used to analyze genetic differences within and among species and even across families [10,11,12]. Cytochrome c oxidase subunit I (COI), as a DNA barcode marker, is widely applied in species identification due to its concise sequence and richness in phylogenetic signals [13,14]. These three molecular markers experience different selective pressures and exhibit varied evolutionary rates, enabling them to provide diverse genetic information. The integrated use of these three genetic markers can comprehensively reveal the genetic structure and evolutionary history of a species, providing an important foundation for population management.

The alpine musk deer (*Moschus chrysogaster*) is distributed across the Tibetan Plateau and its surrounding mountains [15,16]. It is a renowned economic animal, with males possessing scent glands in their abdomens that secrete musk. In recent decades, habitat degradation and loss in China, coupled with illegal poaching, have led to a sharp decline in population numbers, particularly in the forested areas of the peripheral mountains of the Tibetan Plateau [17,18]. In 1990, China initiated the domestication of alpine musk deer in the Xinglong Mountains of Gansu Province [19], establishing the world’s only breeding base for this species. The aim is to breed the population while serving the dual goals of musk production and rewilding. For both objectives, the genetic diversity of the alpine musk deer population is the most important indicator of high-quality germplasm resources. To date, the breeding base has conducted research on the effects of artificial musk extraction on the behavior and physiology of captive alpine musk deer [20], the relationship between aggression, fecal steroid hormone levels, and musk secretion [21], as well as the determination of physiological and biochemical normal values [22]. There is still limited research on the conservation genetics of wild alpine musk deer, with only studies on genetic diversity based on *Cytb* markers [23] and mitochondrial genomics [24]. However, since the establishment of artificial breeding 35 years ago, there has been no study or evaluation of the genetic diversity of captive alpine musk deer. This not only limits scientific assessments of their long-term survival and rewilding potential but also leaves uncertainties in genetic management and breeding strategies. Therefore, this study aims to systematically analyze the genetic diversity of captive alpine musk deer in Gansu using three markers—mtDNA D-Loop, *Cytb*, and COI—and to compare this data with that of wild populations, providing theoretical support for future population genetic management and conservation measures.

## 2. Materials and Methods

### 2.1. Collection of Fecal Samples from Captive Alpine Musk Deer

Samples for this study were collected at the Fengchun Alpine Musk Deer Breeding Base in the Xinglong Mountains of Gansu Province. The base is located in Yuzhong County, Lanzhou City, at an elevation of 2350 m. It falls within a continental semi-arid climate zone, with an annual precipitation of 428 mm and an average annual temperature of 7.5 °C, reaching a maximum of 19 °C in the hottest month and a minimum of −7.9 °C in the coldest month. The breeding base houses over 2000 alpine musk deer, utilizing a breeding model of 1 male to 4 females. The breeding group has a communal activity area of approximately 60 square meters, with each deer provided a separate small shelter. Fecal samples for this study were collected in March 2024, totaling 409 fresh samples. Collection occurred between 5:00 and 6:00 a.m. The samples were placed in sterile zip-lock bags, labeled with ear tag numbers, sex, and sampling time. They were temporarily stored in a portable refrigerator and shipped back to the laboratory using dry ice, where they were subsequently stored at −20 °C until DNA extraction could be performed.

### 2.2. Amplification of Mitochondrial Genes in Captive Alpine Musk Deer

#### 2.2.1. DNA Extraction

The experimental methods were adapted from Piggott [25] and Tang Jie et al. [26], with appropriate modifications. The specific steps for processing fecal samples and extracting DNA are as follows:

1. Prepare cell suspension from fecal samples. Approximately 0.5 g of fecal sample (about 5 pellets) was placed in a 15 mL centrifuge tube. Then, 3 mL of a buffer solution mixed with PBS and SDS [27] was added. The mixture was allowed to stand for 5 min, after which it was vigorously vortexed for 30–45 s. The entire liquid was then transferred to a new 15 mL centrifuge tube and centrifuged at 2000 rpm for 10 min. The supernatant was discarded, and the pellet was resuspended in 0.2 mL of PBS buffer. After resuspension, the mixture was transferred to a 1.5 mL centrifuge tube. This process effectively prepared a cell suspension containing the intestinal epithelial cells of the alpine musk deer from the fecal sample.

2. Extract genomic DNA from the alpine musk deer using a blood/cell/tissue genomic DNA extraction kit (TianGen) (Beijing, China) according to the manufacturer’s instructions.

#### 2.2.2. PCR Amplification and Sequencing

The primers for amplifying the mtDNA D-Loop region sequence were designed by Sangon Biotech (Beijing, China) after downloading the target sequence from NCBI (accession number AY835379.1). The primers for amplifying the mtDNA *Cytb* region sequence were designed by Sangon Biotech after downloading the target sequence from NCBI (accession number AF026887.1). The primers for amplifying the mtDNA COI region sequence were designed by Sangon Biotech after downloading the target sequence from NCBI (accession number NC_020093.1), and the specific primer information is shown in Table 1.

The PCR amplification volume was 25 μL, including 12.5 μL 2 ×Es Taq PCR Master Mix (Dye), 3 μL DNA template, 4 μL 2%BSA, 1 μL forward primer, 1 μL reverse primer, and 3.5 μL ddH2O. The conditions were performed as follows: initial denaturation at 94 °C for 5 min, followed by 34 cycles of PCR (denaturation at 94 °C for 30 s, annealing at 57 °C for 30 s, and extension at 72 °C for 1 min), and a final extension of 72 °C for 8 min. PCR products were examined by electrophoresis on a 1.0% agarose gel. We observed whether bands were present and if their sizes matched the expected values. Qualified PCR products were sent to a biotechnology company (Sangon Biotech) (Beijing, China) for purification and Sanger sequencing.

#### 2.2.3. Acquisition of Mitochondrial Data from Wild Alpine Musk Deer

To systematically evaluate the levels of genetic diversity in captive alpine musk deer and explore their potential for long-term husbandry and rewilding, this study selected publicly available genetic sequences of wild alpine musk deer for comparison. We downloaded 48 mtDNA D-Loop sequences from NCBI, categorized into two populations based on their origins: India (designated as YD_D, accession numbers: PP429508-12) and China (designated as ZG_D, accession numbers: AY835337-79). Additionally, we downloaded 8 mtDNA *Cytb* sequences, classified into three populations: Gansu, China (designated as ZG1, accession numbers: AB019636-39); Sichuan, China (designated as ZG2, accession number: AF026887); and India (designated as YD, accession numbers: AY684631, AY245525, AY395686). Furthermore, we obtained 6 complete mitochondrial genome sequences, divided into two populations based on their origins: Gansu, China (designated as ZG3, accession numbers: KC425457, KP684123); and Sichuan, China (designated as ZG4, accession numbers: NC_020093, JQ608470, MK697349, MW284875). This comprehensive dataset facilitated a robust analysis of genetic diversity between captive and wild populations of alpine musk deer. In addition, the captive alpine musk deer population in this paper is referred to as JY.

### 2.3. Data Analysis

#### 2.3.1. Phylogenetic Tree Construction

The obtained forward and reverse sequences were assembled using SeqMan 7.1.0.44 software [28] and manually corrected. All sequences were aligned using MEGA [29,30,31]. After importing the sequences, the Clustal W program was selected for alignment. To avoid issues such as missed reads, excess reads, and assembly errors during the bidirectional sequencing process, the detected missing, insertion, and variation sites were rechecked against the sequencing chromatograms, and manual corrections were made [32]. Using MEGA software, the nucleotide composition of the final retained homologous region sequences was calculated, and transition and transversion sites were detected to compute the genetic distance matrix among samples. A phylogenetic tree was constructed using the neighbor-joining (NJ) method, with the bootstrap method repeated 1000 times.

#### 2.3.2. Haplotype Network Construction, Polymorphism Analysis

We imported the aligned raw sequences into DnaSP6 software. In DnaSP, we designated all sequences as a single population and exported the grouped data as two files: arp and hap. We opened the arp and hap files in a text editor, and merged the information from both files into one Nexus file. Finally, we used PopART 1.7 software [33] to construct haplotype networks based on the mitochondrial D-Loop region, *Cytb*, COI, and combined sequences. Using DnaSP 6 [34], we calculated the number of variable sites, haplotypes (h), nucleotide diversity (Pi), haplotype diversity (Hd), average nucleotide differences (K), and mismatch distribution for each population.

#### 2.3.3. Genetic Differentiation Coefficient and Molecular Variance Analysis Methods

The genetic differentiation coefficient (*F_ST_*) and analysis of molecular variance (AMOVA) were conducted using Arlequin 3.5 [35]. In the significance testing, a *p*-value > 0.05 indicates that the differences are not significant, a *p*-value < 0.05 indicates that the differences are significant, and a *p*-value < 0.01 indicates that the differences are highly significant.

#### 2.3.4. Richness Estimation

EstimateS 9.1.0 [36,37] was used to estimate the total number of haplotypes based on the classic Chao 1 richness estimator, with a 95% confidence interval (95% CL) calculated through 1000 permutations. A haplotype accumulation curve was constructed with the number of individuals as the x-axis and haplotype number as the y-axis.

#### 2.3.5. Sequence Concatenation

PhyloSuite was used to concatenate the D-Loop, *Cytb*, and COI sequences. The concatenated sequences typically carry more phylogenetic information than single-gene sequences, facilitating the generation of a more stable phylogenetic tree with higher support values [38].

## 3. Results

### 3.1. D-Loop Gene Sequence and Haplotype

After concatenation and multiple sequence alignment, a total of 386 alpine musk deer mtDNA D-Loop sequences were obtained, with an effective analysis sequence length of 505 bp. The base composition exhibited a typical A + T bias, with an average A + T content of 65.9%, which is higher than the average G + C content of 34.1%. The G content was the lowest, consistent with the general characteristics of mitochondrial base composition in vertebrates. There were 482 conserved sites and 23 polymorphic sites, including 1 single nucleotide polymorphism and 22 informative sites. The nucleotide diversity (Pi) was 0.01073, and indicating that the average number of nucleotide differences (K) was 5.417. and haplotype diversity (Hd) was 0.639.

Based on the mtDNA D-Loop data of alpine musk deer, a total of 19 haplotypes were defined among 386 individuals. The estimated haplotype richness (Chao 1) was 63. The genetic distance between haplotypes ranged from 0.002 to 0.024. Hap4 was the most frequently occurring haplotype, shared by 220 individuals, accounting for approximately 56.99%. Hap2 and Hap6 were also relatively common, representing 12.43% and 12.17% of all individuals, respectively. Additionally, 11 haplotypes were rare, appearing only once.

**Figure 1:**
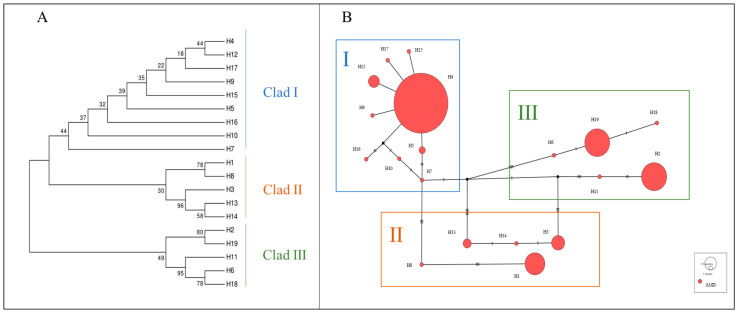
(**A**) NJ tree of alpine musk deer mtDNA D-Loop haplotypes. (**B**) Network diagram of alpine musk deer mtDNA D-Loop haplotypes.

Based on genetic distances, a haplotype phylogenetic tree (NJ tree) was constructed using the neighbor-joining method. All haplotypes were divided into three branches, with significant size differences among them, each including rare haplotypes (Figure 1A). Haplotype group I contained the most haplotypes, with nine in total, making it the dominant group, while both haplotype groups II and III each had five haplotypes.

The haplotype median-joining network constructed using the maximum parsimony method visually illustrates the phylogenetic relationships among haplotypes (Figure 1B). This result is consistent with the clustering observed in the NJ tree, where black represents missing haplotypes.

### 3.2. COI Sequence and Haplotype

After concatenation and multiple sequence alignment, a total of 407 alpine musk deer mtDNA COI sequences were obtained, with an effective analysis sequence length of 534 bp. The base composition exhibited a typical A + T bias, with an average A + T content of 58.7%, which is higher than the average G + C content of 41.3%. The G content was the lowest, consistent with the general characteristics of mitochondrial base composition in vertebrates.

There were 526 conserved sites and 8 polymorphic sites, including 2 single nucleotide polymorphisms and 6 informative sites. The nucleotide diversity (Pi) was 0.00196, and the average number of nucleotide differences (K) was 1.044. A total of nine haplotypes were defined among the 407 individuals, with a haplotype diversity (Hd) of 0.474.

### 3.3. Cytb Sequence and Haplotype

After concatenation and multiple sequence alignment, a total of 409 alpine musk deer mtDNA *Cytb* sequences were obtained, with an effective analysis sequence length of 987 bp. The base composition exhibited a typical A + T bias, with an average A + T content of 59.5%, which is higher than the average G + C content of 40.5%. The G content was the lowest, consistent with the general characteristics of mitochondrial base composition in vertebrates.

There were 976 conserved sites and 11 informative sites. The nucleotide diversity (Pi) was 0.00264, and the average number of nucleotide differences (K) was 2.602. A total of 14 haplotypes were defined among the 409 individuals, with a haplotype diversity (Hd) of 0.612.

### 3.4. Phylogenetic and Network Relationship Analysis

Based on the combined data of mtDNA D-Loop, *Cytb*, and COI from 338 individuals, a total effective analysis sequence length of 2027 bp was obtained after concatenation and multiple sequence alignment. The base composition exhibited a typical A + T bias, with an average A + T content of 60.9%, which is higher than the average G + C content of 39.1%. The G content was the lowest, consistent with the general characteristics of mitochondrial base composition in vertebrates.

There were 1985 conserved sites and 42 polymorphic sites. Among the polymorphic sites, there were three two-variant sites: 1308, 1516, and 1532. Additionally, there were 39 informative sites with two variants. The nucleotide diversity (Pi) was 0.00445, and the average number of nucleotide differences (K) was 9.019. The haplotype diversity (Hd) was 0.922.

Based on the combined data of mtDNA D-Loop, *Cytb*, and COI, a total of 95 haplotypes were defined among 338 alpine musk deer individuals. The estimated haplotype richness (Chao1) was 182. The genetic distance between haplotypes ranged from 0.0005 to 0.0115. Hap3 was the most frequently occurring haplotype, shared by 85 individuals, accounting for approximately 25.15%. Additionally, 37 haplotypes were rare, appearing only once.

**Figure 2:**
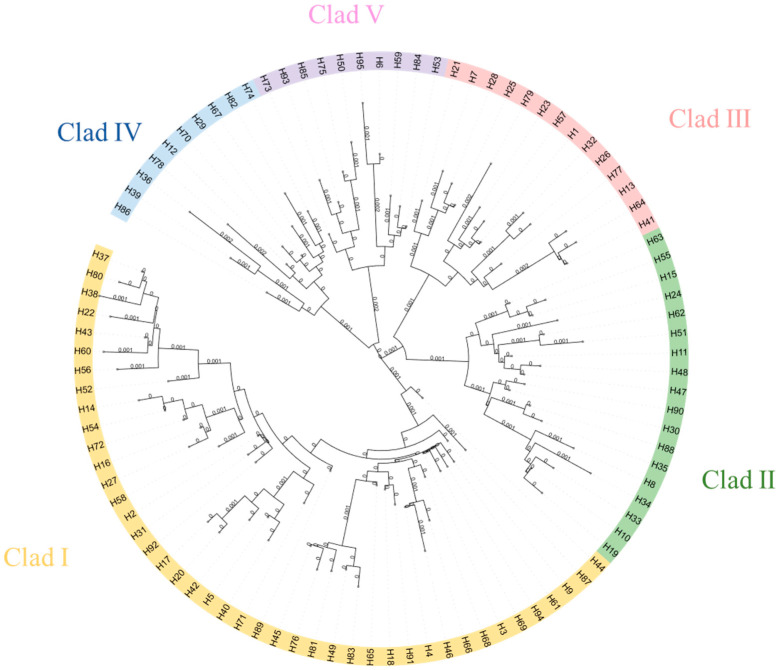
NJ tree of alpine musk deer haplotypes based on combined sequences.

Using the neighbor-joining method based on genetic distances, a haplotype phylogenetic tree (NJ tree) was constructed, dividing all haplotypes into five branches, with significant size differences among them, each including rare haplotypes (Figure 2). Haplotype group I contained the highest number of haplotypes, making it the dominant group. Within this group, H3 had the highest frequency and gave rise to the most derived haplotypes, suggesting it may be the ancestral haplotype of the population. The other haplotypes exhibited one or more mutations and were connected to the main haplotypes, displaying a clear “radiative” topology.

**Figure 3:**
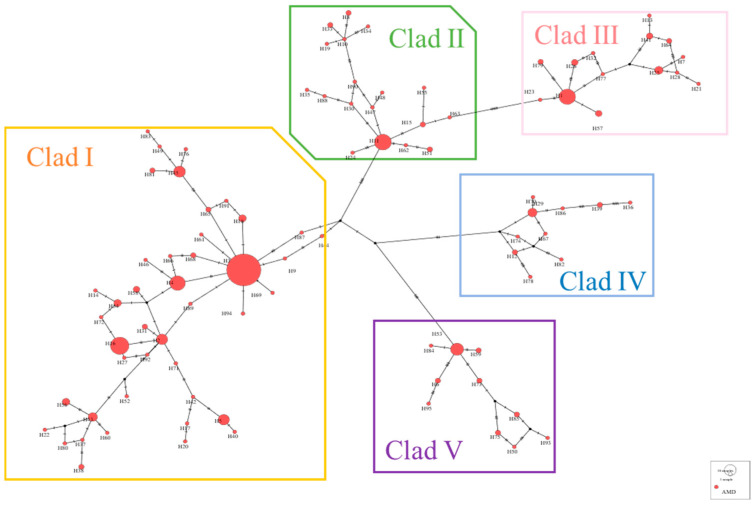
Network diagram of alpine musk deer haplotypes based on combined sequences.

A haplotype median-joining network constructed using the maximum parsimony method visually illustrates the phylogenetic relationships among haplotypes (Figure 3). This result is consistent with the clustering observed in the NJ tree, where black represents missing haplotypes.

### 3.5. Comparison of Genetic Diversity Between Captive and Wild Alpine Musk Deer

#### 3.5.1. Comparison of Genetic Diversity Based on *Cytb*

Organize the data from the three populations ZG1, YD, and ZG4 (selecting populations with a sample size ≥3 to ensure the data is meaningful), along with data from wild musk deer in western China [23] and captive musk deer populations. The obtained data results are shown in Table 2.

**Figure 4:**
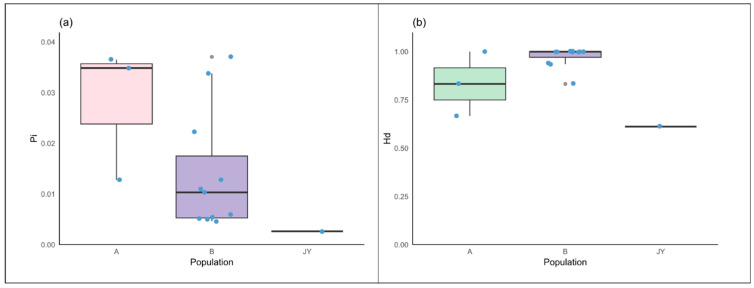
(**a**) Comparison of nucleotide diversity. (**b**) Comparison of haplotype diversity. Note: For statistical convenience, the populations ZG1, YD, and ZG4 are grouped as class A, while the data from wild alpine musk deer in western China are grouped as class B.

We compared and organized these data, and the comparison results are illustrated in Figure 4.

From the figure, it is evident that captive alpine musk deer exhibit lower genetic diversity and haplotype diversity compared to wild populations.

The analysis of data from captive and wild alpine musk deer populations is summarized in Table 3, which shows the genetic differentiation among various groups. The genetic differentiation analysis indicates that the JY population is highly differentiated from ZG1, YD, and ZG4, with genetic differentiation coefficients (*Fst*) greater than 0.25.

As shown in Table 4, the analysis of molecular variance (AMOVA) includes variance among populations (Va) and within populations (Vb). The results indicate that 82.88% of the variation is attributed to differences among populations, while 17.12% of the genetic variation arises within populations. Overall, the genetic differentiation among populations is highly significant, with a fixation index of 0.82884.

**Figure 5:**
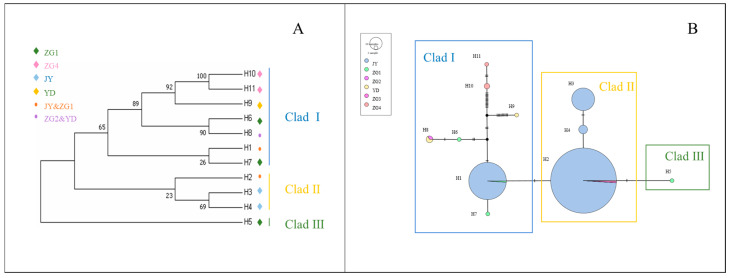
(**A**) Neighbor-joining (NJ) tree of mtDNA *Cytb* haplotypes for wild and captive alpine musk deer. (**B**) Network diagram of mtDNA *Cytb* haplotypes for captive and wild alpine musk deer.

The mtDNA *Cytb* data from captive alpine musk deer and the populations ZG1, ZG2, ZG3, ZG4, and YD were integrated, resulting in 11 defined haplotypes from 423 individuals. Based on genetic distance, a neighbor-joining (NJ) tree was constructed, dividing all haplotypes into three branches, each with significant size differences, including rare haplotypes (Figure 5A). The captive population shares haplotypes Hap1 and Hap2 with the ZG1 population, while ZG2 and YD share haplotype Hap8. A haplotype median network constructed using the maximum parsimony method visually illustrates the phylogenetic relationships among haplotypes (Figure 5B). This result is consistent with the clustering observed in the NJ tree, with black indicating missing haplotypes.

From Figure 5A, it can be observed that captive alpine musk deer primarily cluster on one evolutionary branch, and the populations do not form a monophyletic group.

Figure 5B shows that haplotypes Hap1 and Hap2 represent the main evolutionary direction. Although there are genetic differences among the populations, these differences are relatively small, consistent with the NJ tree results. This confirms the existence of gene flow among the populations.

#### 3.5.2. Comparison of Genetic Diversity Based on D-Loop

The analysis compares the captive alpine musk deer population with four other populations: ZG_D, YD, ZG3, and ZG4. Table 5 presents the genetic diversity parameters for each population.

It can be observed from the table that, compared to wild alpine musk deer, the captive population exhibits lower genetic diversity and haplotype diversity.

An analysis of the data between the captive population and wild populations is presented in Table 6, which shows genetic differentiation based on the D-Loop region. The genetic differentiation analysis indicates that YD_D is highly differentiated from ZG4, and JY is highly differentiated from YD_D and ZG4. Additionally, ZG4 is highly differentiated from ZG_D and ZG3, with *Fst* values exceeding 0.25. A few other populations also show moderate differentiation.

As shown in Table 7, the analysis of molecular variance (AMOVA) includes among-population variance (Va) and within-population variance (Vb). The results indicate that 39.64% of the genetic variation arises from between-population differences, while 60.36% is attributed to genetic variation within populations. Overall, the genetic differentiation among populations is highly significant, with a fixation index of 0.39638.

**Figure 6:**
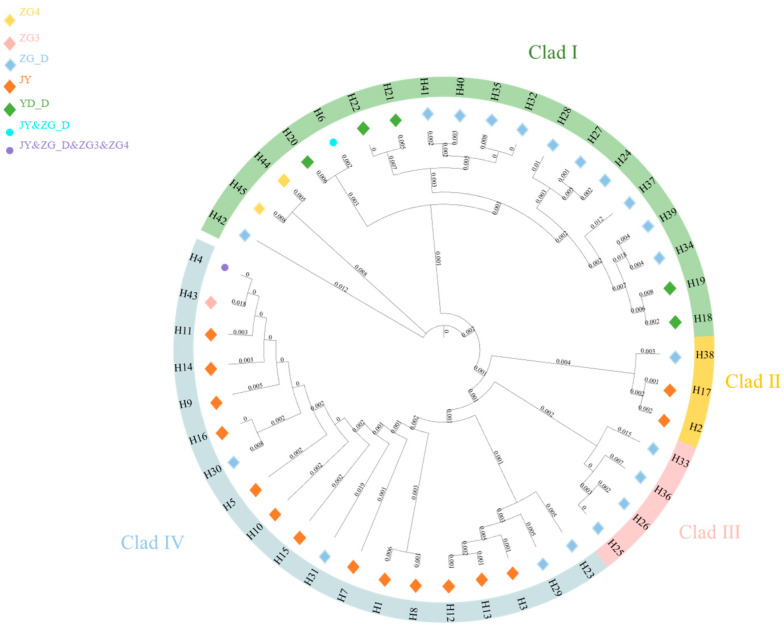
Neighbor-joining (NJ) tree of mtDNA D-Loop haplotypes for wild and captive alpine musk deer.

**Figure 7:**
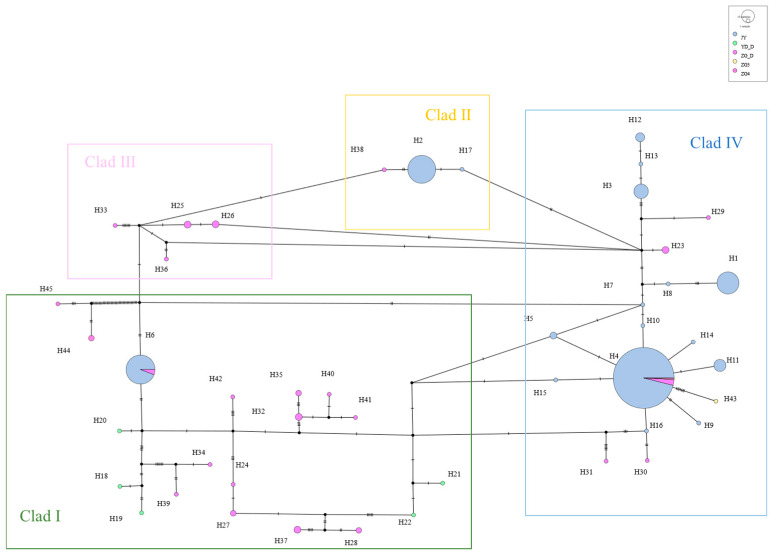
Network diagram of mtDNA D-Loop haplotypes for captive and wild alpine musk deer.

The mtDNA D-Loop data from captive alpine musk deer and the populations ZG_D, ZG3, ZG4, and YD_D were integrated, resulting in 45 defined haplotypes from 440 individuals. Based on genetic distance, a neighbor-joining (NJ) tree was constructed, categorizing all haplotypes into four branches, with significant size differences, each including rare haplotypes (Figure 6). Among these, the captive population shares haplotype Hap6 with ZG_D and haplotype Hap4 with ZG_D, ZG3, and ZG4. A haplotype median network constructed using the maximum parsimony method visually illustrates the phylogenetic relationships among haplotypes (Figure 7). This result is consistent with the clustering observed in the NJ tree, with black indicating missing haplotypes. From Figure 6, it can be seen that captive alpine musk deer primarily cluster in two evolutionary branches, while wild populations are dispersed across four haplogroups, indicating that the populations do not form a monophyletic group.

From Figure 7, it can be observed that haplotype Hap4 represents the main evolutionary direction and is shared among all populations. In contrast, other evolutionary branches show varying degrees of decline. This phenomenon may be due to the advantageous traits of Hap4 that enhance survival in the wild and meet human needs. During breeding, selection was applied; however, the lack of molecular breeding techniques or scientific husbandry methods may have contributed to the observed decline in genetic diversity. This result serves as a warning, emphasizing the need for appropriate methods to protect the alpine musk deer, an endangered economic species.

## 4. Discussion

The alpine musk deer, a national Class I protected species, is highly valued for producing high-quality musk, which has significant medicinal and ecological value. Its wild population has sharply declined due to poaching and habitat destruction. Since 1990, captive breeding programs have been initiated in Gansu, China, to meet the demand for musk and to protect the genetic resources of wild populations. While captive breeding programs are the cornerstone of modern species conservation, such measures often bring about unforeseen genetic consequences. Due to the limited number of founding individuals and factors like genetic drift, endangered species in captive environments may face a continuous loss of genetic diversity.

To assess this effect, we compared the mitochondrial DNA (mtDNA) variation between wild and captive alpine musk deer populations.

We collected 409 fresh fecal samples from the breeding base in Xinglong Mountain, Gansu Province, and sequenced the highly variable control region (D-Loop) [6,9] along with two protein-coding genes (cytochrome b [*Cytb*] and cytochrome c oxidase I [COI]) [10,11,12,13,14], obtaining 386, 409, and 407 sequences, respectively. Based on these sequences, we calculated genetic indicators such as polymorphic sites, the number of haplotypes, haplotype diversity (Hd), nucleotide diversity (π), and interspecific *F_ST_*. The results showed significant differences in genetic diversity between captive and wild populations. Compared to wild populations, the nucleotide diversity (Pi) and haplotype diversity (Hd) in captive populations were significantly lower, indicating a severe loss of genetic variation during captivity. The *F_ST_* values between captive and wild populations were both greater than 0.25, suggesting substantial genetic differentiation after 35 years of artificial breeding. Wild populations exhibited a large number of unique mtDNA haplotypes and higher nucleotide diversity, while captive populations contained only a few haplotypes, resulting in extremely low overall diversity. Specifically, multiple distinct haplotypes were detected among wild individuals, whereas captive individuals were highly homogeneous, clustering around a limited number of haplotypes. The haplotype network and phylogenetic tree based on the D-Loop showed that captive populations concentrated around a few dominant haplotypes (e.g., Hap4), with a high proportion of rare haplotypes. The haplotypes in captive populations formed a limited lineage distinctly separated from the wild gene pool, consistent with the founder effect and genetic drift caused by inbreeding. Additionally, some haplotypes (e.g., Hap4) were shared between captive and wild populations, likely due to contributions from wild individuals captured during the early stages of breeding. However, the lack of new genetic input during long-term captivity exacerbated the decline in diversity, resulting in captive populations retaining only a small fraction of the ancestral genetic diversity of the species.

The findings underscore the risk that captive breeding may not capture the full genetic breadth of a species. Maintaining genetic diversity is a crucial objective in conservation biology, and this study suggests that relying solely on captive populations may undermine the achievement of this goal. In particular, the significant founder effect and genetic drift observed during captivity are likely to lead to a loss of genetic diversity and abnormal lineage differentiation. This loss of variability could weaken the population’s adaptive potential and affect the success of future reintroduction efforts into the wild.

This study analyzes the mtDNA data of captive alpine musk deer in Gansu, revealing the genetic diversity characteristics of the population after 35 years of management. mtDNA exhibits strict maternal inheritance, meaning individuals from the same maternal lineage share the same haplotype. Numerous studies have demonstrated that the mtDNA D-Loop is an effective maternal tracing marker, used to validate recorded maternal lineages [39,40]. In populations, haplotypes that are frequently shared and widely distributed are often considered ancestral haplotypes [41]. The haplotype network can visually illustrate the evolutionary relationships among haplotypes, with the most central and frequent haplotypes regarded as ancestral. Additionally, when the haplotype network displays a star-like structure, it indicates that the population has recently undergone rapid expansion [42,43]. In this study, 19 haplotypes were identified from 386 individuals based on the D-Loop gene, with Chao1 estimating 65 founding maternal lineages. Certain haplotypes (such as Hap4) are dominant, while rare haplotypes are present, indicating a rich but uneven maternal foundation.

Based on the D-Loop analysis, the nucleotide diversity (π) and haplotype diversity (Hd) indices show that the overall genetic diversity of the captive population is lower than that of wild populations. Compared to domesticated forest musk deer (*Moschus berezovskii*) populations in China [44], the alpine musk deer population exhibits higher haplotype diversity but lower nucleotide diversity than the captive forest musk deer. In the haplotype network constructed using the mitochondrial D-Loop region, Hap4 and Hap6 occupy the center, representing the ancestral haplotypes shared among different populations, from which other haplotypes further derive. There is evidence of varying degrees of gene flow among these haplotypes, indicating a clear lineage structure. This situation may be related to the self-breeding and inbreeding present in captive alpine musk deer. Although breeding efforts have become increasingly scientific in recent years, there remains a significant issue of insufficient genetic exchange with wild sources.

In the haplotype network constructed from a partial sequence of the *Cytb* gene, Hap1 and Hap2 occupy the center of the network, primarily consisting of captive alpine musk deer samples. These can be considered ancestral haplotypes for this gene, from which other haplotypes have derived, with these haplotypes largely unique to different populations. Additionally, genetic differentiation analysis revealed that the *F_ST_* values between different populations based on *Cytb* range from −1.0000 to −0.92278, while the *F_ST_* values based on the D-Loop range from 0.15723 to 0.82610. This indicates significant genetic differentiation between captive and wild populations of alpine musk deer. The high level of genetic differentiation between captive and wild populations further confirms the distinct separation of captive haplotypes from the wild gene pool. Analysis of the AMOVA results based on *Cytb* shows that the genetic variation among populations accounts for a substantial 82.88%, further supporting the significant genetic differentiation between captive and wild populations. This differentiation arises from two main factors: first, the lack of continuous introduction of new genetic sources into the captive population, creating a long-standing barrier to gene flow with wild populations; second, the captive breeding model (1 male: 4 females) restricts mating opportunities and promotes inbreeding, leading to genetic drift and a gradual homogenization of genotypes. If such long-term captivity continues without scientific genetic management, it could result in a severe decline in the genetic diversity of alpine musk deer. Therefore, there is an urgent need to establish scientific, effective, and actionable strategies for managing genetic diversity.

This study analyzed the haplotype diversity and nucleotide diversity of three mtDNA segments (COI, *Cytb*, and D-Loop) in alpine musk deer populations, revealing their genetic history. The results indicate that the population underwent rapid expansion following the breeding of a small number of ancestral groups. Haplotype network analysis also shows signs of this rapid expansion. Additionally, comparisons with wild populations reveal that the nucleotide diversity and haplotype diversity of captive populations are lower than those of wild populations, although haplotype diversity is higher than that of forest musk deer. These findings are significant for theories in animal genetics and conservation biology. Firstly, the genetic diversity characteristics align with classic population expansion models, indicating that captive alpine musk deer experienced typical founder effects and rapid expansion. Secondly, the retention of 65 founding maternal lineages highlights the critical role of maternal inheritance in maintaining population diversity. These results enrich the theoretical foundation for the genetic management of rare captive animals and contribute to understanding the history of captive breeding and population evolutionary trends.

Comparing captive populations with wild musk deer populations allows for the assessment of the extent of diversity loss during breeding. In wild forest musk deer, genetic diversity varies by region. For example, the wild forest musk population in Shanxi Province has a haplotype diversity (Hd) of about 0.916 and nucleotide diversity (π) of 0.01505 [45]. In contrast, the captive forest musk deer in Shaanxi have an Hd of approximately 0.908 [44], indicating that the maternal diversity in captive populations has largely maintained the levels of wild sources. However, some wild forest musk populations in the southwestern region exhibit an Hd of only about 0.72 [45], highlighting genetic variation differences within wild populations. For the alpine musk deer (*M. chrysogaster*), wild populations are distributed in high-altitude areas and are sparse, likely experiencing significant bottlenecks, resulting in relatively low genetic diversity. Overall, the genetic diversity of captive alpine musk deer is not higher than the lower limits observed in wild deer. If the captive population originates from a limited genetic pool, its diversity may fall below that of wild populations; conversely, if multiple founding groups are collected, the diversity could be comparable to that of wild populations. Existing studies indicate that captive forest musk deer populations sourced from multiple locations exhibit a higher Hd (up to 0.93) [45], while those from a single source tend to have lower diversity. Therefore, for captive alpine musk deer, the level of diversity should be assessed in conjunction with the size of the founding gene pool and the history of translocation.

In captive breeding systems, the lack of free mating between different breeding bases or lineages often leads to restricted gene flow. Our analysis is consistent with existing studies, showing clear differentiation among captive musk deer populations and relatively low gene exchange. For instance, research on three artificially bred forest musk deer populations in Sichuan indicates that there is only a very low level of gene exchange between populations. Similarly, RAD-seq studies have found moderate differentiation among different captive populations, with minimal gene flow [46,47]. The artificial selection pressures in domesticated environments primarily target productive traits (such as musk yield) or behavioral traits, while mitochondrial DNA is mostly a neutral marker. Therefore, its variation mainly reflects population history and drift. In our data, the observed lineage and genotype frequencies are more likely due to the initial collection range during the early stages of captivity and subsequent population management (such as inbreeding and lineage continuation), rather than direct adaptive selection for domestication.

Insufficient genetic diversity can weaken a species’ ability to respond to environmental changes and diseases, increasing the risk of extinction. For example, studies have reported that captive forest musk deer populations commonly experience fatal diseases, with mortality rates significantly correlated with the severity of inbreeding within the population. In forest musk deer, low diversity and a tendency toward inbreeding have led to issues such as chromosomal defects and decreased immunity [48,49]. Research by Fan et al. [47] further indicates a significant loss of heterozygosity in captive forest musk deer populations, suggesting high levels of inbreeding and potential declines in adaptability. The widespread distribution of haplotypes in wild populations (such as 26 haplotypes in ZG_D) reflects strong evolutionary and adaptive potential, while the more uniform branches in captive populations (e.g., five haplogroups in combined sequences) imply increased inbreeding risks. Overall, if captive alpine musk deer populations continue to lack effective diversity management, they may face adverse consequences such as reduced reproductive capacity, lower offspring survival rates, and increased sensitivity to diseases and environmental stressors. Drawing on experiences from other species within the Cervidae family, maintaining sufficient genetic diversity is crucial for ensuring long-term population health and sustainability.

## 5. Conclusions

Based on the research findings, we discovered that after 35 years of captivity, the genetic diversity of alpine musk deer populations has significantly declined. To improve the genetic quality of captive populations, a multifaceted management approach should be adopted. Firstly, the effective breeding population size should be expanded by introducing new founding individuals to enhance the genetic base while avoiding excessive inbreeding. If necessary, individuals can be sourced from different geographical locations (such as multiple wild populations or breeding centers) to enrich the gene pool. Secondly, it is essential to establish and improve pedigree records to document individual relationships, aiming to avoid inbreeding during pairings. Thirdly, moderate exchanges of breeding musk deer between different breeding bases can facilitate gene flow, helping to balance genetic differences among populations. Additionally, molecular genetic methods should be employed to regularly monitor genetic diversity and the genetic status of offspring. Timely adjustments to management strategies should be made if a decline in diversity or trends of inbreeding are detected. Finally, captive breeding should be integrated with wild conservation efforts. Protecting the natural habitats and wild populations of alpine musk deer will create conditions for potential genetic supplementation and future reintroductions. Through these comprehensive measures, the genetic diversity of captive alpine musk deer can be effectively enhanced, thereby strengthening the long-term viability and rewilding potential of the population.

## Figures and Tables

**Table 1:** Amplification primers.

Locus	Primer Sequences (5′-3′)	Target Fragment (bp)
D-Loop	F: CCCTAAGACTCAAGGAAGAAGC R: TACCCCCACAGATTATGGGCC	530
*Cytb*	F: ATGATCAATATTCGAAAATCTCACCC R: TCTTCATTTTAGGAGATTATTTTCAATTT	1140
COI	F: TTGAAGCYGGAGCAGGAACAG R: AARTAGGCTCGTGTGTCRAC	561

**Table 2:** Genetic diversity comparison table based on *Cytb.*

Population	Sample Size (n)	Number of Variable Sites	Number of Haplotypes (H)	Nucleotide Diversity (Pi)	Haplotype Diversity (Hd)	Average Number of Nucleotide Differences (K)
ZG1	4	9	4	0.01278	1.000	4.500
ZG4	4	83	3	0.03655	0.833	41.667
YD	3	17	2	0.03487	0.667	11.333
Aba county *	5	12	5	0.00464	1.000	4.800
Muli county *	5	13	5	0.00503	1.000	5.200
Baiyu county *	4	10	4	0.00516	1.000	5.333
Yajiang county *	4	21	3	0.01032	0.833	10.667
Ganzi county *	6	33	6	0.01103	1.000	11.400
Luhuo county *	16	92	12	0.03380	0.942	34.883
Dege county *	23	49	17	0.01278	0.935	13.217
Shimian county *	4	76	4	0.03707	1.000	38.333
Zhangye city *	3	23	2	0.02224	1.000	23.000
Gongbujiangda county *	4	12	4	0.00596	1.000	6.167
Cangdu county *	5	14	5	0.00542	1.000	5.600
JY	409	11	14	0.00264	0.612	2.602

Note: The content indicated by * is from Wild Musk Deer in Western China [23]. JY refers to the captive alpine musk deer in this study.

**Table 3:** Genetic differentiation index among alpine musk deer populations based on *Cytb.*

Population	JY	ZG1	ZG2	YD	ZG3	ZG4
JY						
ZG1	0.42977 (+)					
ZG2	0.086429 (−)	0.25000 (−)				
YD	0.85017 (+)	0.17207 (−)	−1.00000 (−)			
ZG3	−0.18146 (−)	0.01370 (−)	1.00000 (−)	0.23982 (−)		
ZG4	0.92278 (+)	0.53398 (−)	0.35714 (−)	0.41640 (−)	0.49415 (−)	

Note: (+) indicates a *p*-value < 0.05; (−) indicates a *p*-value > 0.05.

**Table 4:** Molecular variance analysis (AMOVA) of alpine musk deer populations based on *Cytb.*

Source of Variation	Degrees of Freedom	Sum of Squares	Variance Components	Percentage of Variation
Among populations	5	54.381	1.91075 Va	82.88
Within populations	417	164.541	0.39458 Vb	17.12
Total	422	218.922	2.30534	
Fixation Index	0.82884 (++)			

Note: (++) indicates a *p*-value < 0.01.

**Table 5:** Genetic diversity comparison table based on D-Loop.

Population	Sample Size (n)	Number of Variable Sites	Number of Haplotypes (H)	Nucleotide Diversity (Pi)	Haplotype Diversity (Hd)	Average Number of Nucleotide Differences (K)
ZG_D	43	60	26	0.02469	0.961	13.083
ZG3	2	7	2	0.01799	1.000	7.000
ZG4	4	44	3	0.05771	0.833	22.333
YD_D	5	16	5	0.02262	1.000	8.800
JY	386	23	19	0.01073	0.639	5.417

Note: JY refers to the captive alpine musk deer in this study.

**Table 6:** Genetic differentiation index among musk deer populations based on D-Loop.

Population	JY	YD-D	ZG-D	ZG3	ZG4
JY					
YD-D	0.54239 (+)				
ZG-D	0.20985 (+)	0.15723 (+)			
ZG3	0.25312 (−)	0.42647 (−)	0.22867 (+)		
ZG4	0.82610 (+)	0.57926 (+)	0.66563 (+)	0.46716 (+)	

Note: (+) indicates a *p*-value < 0.05; (−) indicates a *p*-value > 0.05.

**Table 7:** Molecular variance analysis (AMOVA) of alpine musk deer populations based on D-Loop.

Source of Variation	Degrees of Freedom	Sum of Squares	Variance Components	Percentage of Variation
Among populations	4	160.340	1.55429 Va	39.64
Within populations	435	1029.633	2.36697 Vb	60.36
Total	439	1189.973	3.92126	
Fixation Index	0.39638 (++)			

Note: (++) indicates a *p*-value < 0.01.

## Data Availability

The mtDNA CO| sequence data are available from NCBI, under accession numbers PV668829, PV668871-PV668872, PV668890, PV668892, PV668896-PV668897, PV668899, and PV668900. The mtDNA *Cytb* sequence data are available from NCBI, under accession numbers PV686471-PV686484. The mtDNA D-Loop sequence data are available from NCBI, under accession numbers PV767511-PV767529.
